# Antioxidant Therapeutic Strategies in Neurodegenerative Diseases

**DOI:** 10.3390/ijms23169328

**Published:** 2022-08-19

**Authors:** Constanza Morén, Ruth Mary deSouza, Darly Milena Giraldo, Christopher Uff

**Affiliations:** 1 Centro de Investigación en Red de Enfermedades Raras (CIBERER), 28029 Madrid, Spain; 2 Institut d’Investigacions Biomèdiques August Pi i Sunyer (IDIBAPS), 08036 Barcelona, Spain; 3 Faculty of Medicine, University of Barcelona-Hospital Clínic of Barcelona, 08036 Barcelona, Spain; 4 Emergency Department, Guy’s and St. Thomas Hospital (NHS Foundation Trust), London SE1 9RT, UK; 5 Neurology Department, Hospital Comarcal Sant Jaume de Calella, 08370 Barcelona, Spain; 6 Neurotrauma and Neurovascular Surgery, Royal London Hospital, London E1 1FR, UK

**Keywords:** antioxidants, Alzheimer’s disease, amyotrophic lateral sclerosis, Huntington’s disease, mitochondria, neurodegeneration, Parkinson’s disease, traumatic brain injury

## Abstract

The distinguishing pathogenic features of neurodegenerative diseases include mitochondrial dysfunction and derived reactive oxygen species generation. The neural tissue is highly sensitive to oxidative stress and this is a prominent factor in both chronic and acute neurodegeneration. Based on this, therapeutic strategies using antioxidant molecules towards redox equilibrium have been widely used for the treatment of several brain pathologies. Globally, polyphenols, carotenes and vitamins are among the most typical exogenous antioxidant agents that have been tested in neurodegeneration as adjunctive therapies. However, other types of antioxidants, including hormones, such as the widely used melatonin, are also considered neuroprotective agents and have been used in different neurodegenerative contexts. This review highlights the most relevant mitochondrial antioxidant targets in the main neurodegenerative disorders including Alzheimer’s disease, Parkinson’s disease, and Huntington’s disease and also in the less represented amyotrophic lateral sclerosis, as well as traumatic brain injury, while summarizing the latest randomized placebo-controlled trials.

## 1. Introduction

Neurodegenerative diseases are a heterogeneous group of disorders characterized by the progressive loss of function and death of specific groups of neurons that give rise to the clinical manifestation of the disease. Pathophysiologically, alterations in specific proteins lead to dysfunction of different cellular pathways, including increased numbers of reactive oxygen species (ROS) derived from mitochondrial dysfunction (a main focus of this study), excitotoxicity, synaptic dysfunction, impairment of protein degradation systems, endoplasmic reticulum stress, DNA damage, inflammation, and cell cycle reentry [1]. Their complex interaction makes it difficult to understand the mechanisms that generate neurotoxicity and cell death, as well as to find an effective treatment.

The human brain, typically 2% of normal body weight, receives 15% of the cardiac output and consumes approximately 20% of the total basal oxygen (O_2_^−^) [2]. In line with the hypothesis of a mitochondrial basis in neurodegeneration, oxidative tissues with high energy demand are the most vulnerable to oxidative phosphorylation system (OXPHOS) defects [3]. Furthermore, the most metabolically active parts of the brain (the cortex, in particular the motor cortex and thalami, receiving three times the blood flow of white matter) are most vulnerable to hypoxic ischemic encephalopathy. Based on this, antioxidant therapeutic strategies for the treatment of several brain pathologies in order to restore redox equilibrium by scavenging free ROS are considered a promising approach.

## 2. Mitochondria and Oxidative Stress

The inner mitochondrial membrane, impervious to almost all molecules and ions, is highly folded, shaping the mitochondrial cristae, where the enzymatic complexes of the OXPHOS are embedded [4]. OXPHOS enables the synthesis process of ATP coupled to oxygen consumption, through the transfer of electrons by the five mitochondrial enzymatic complexes. The electrons flow through the mitochondrial respiratory chain through oxidation–reduction (or redox) reactions ending in complex IV, where oxygen is the final receptor for the electrons and is reduced to H_2_O. Thus, in the OXPHOS, oxygen is consumed and an electrochemical gradient is established, driving ATP synthesis [4]. In the case of OXPHOS dysfunction, intermediate reactive metabolites derived from oxygen, so-called ROS may be generated, placing mitochondria as a main source of ROS. These species are free radical and nonradical molecules, with decoupled electrons, and are considered highly oxidizing, unstable and capable of damaging most cellular molecules and structures [4]. Such is the case of superoxide anion (O_2_^−^) and hydrogen peroxide (H_2_O_2_), which are relatively stable, although hydroxyl radical (OH^−^) and peroxynitrite (ONOO^−^) are highly reactive. All of them are derived from O_2_^−^, which is mainly generated in mitochondrial respiratory chain complexes I (CI) and III (CIII) through redox reactions of coenzyme Q (CoQ), which in its semi-reduced form is capable of auto-oxidizing and returning to its oxidized form, transferring an electron and converting molecular oxygen to O_2_^−^ [4]. The brain is highly susceptible to oxidative stress and this is explained by multiple reasons, including unsaturated lipid enrichment, high mitochondrial demand, relevance of calcium metabolism, key role of glutamate, modest antioxidant defense, redox active transition metals and potential neurotransmitter auto-oxidation, as previously shown in the literature [2].

## 3. Enzymatic and Non-Enzymatic Antioxidant Molecules

**Figure 1:**
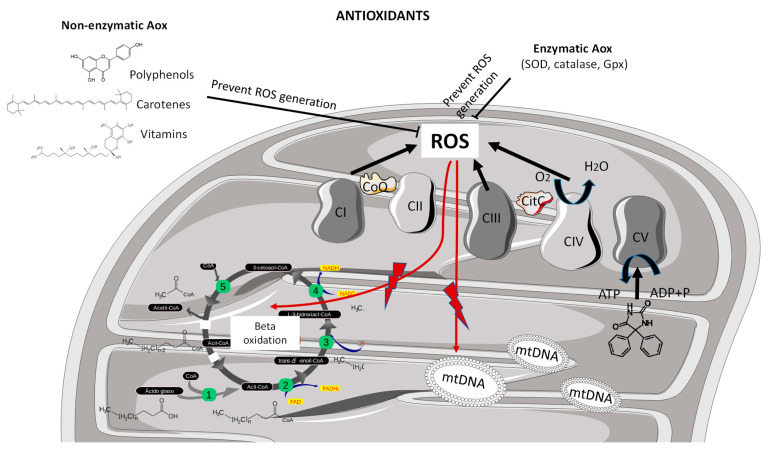
Mitochondrial generation of ROS and antioxidant mechanism of action. Mitochondrial-driven oxidative stress derived from suboptimal function at the level of complex I, complex III and complex IV enzymatic activities of the mitochondrial respiratory chain. Reactive oxygen species may damage different cell structures and molecules including lipids, b-oxidation cycle compounds or nucleic acids such as mitochondrial DNA. Non-enzymatic antioxidants, including polyphenols, carotenes and vitamins, and endogenous enzymatic antioxidants, including superoxide dismutase, catalase and glutathione peroxidase, exert protective mechanisms by scavenging the ROS towards redox equilibrium. ADP, adenosine diphosphate; Aox, antioxidant; ATP, adenosine triphosphate; CI, complex I; CII, complex II; CIII, complex III; CIV, complex IV; CV, complex V; Gpx, glutathione peroxidase; mtDNA, mitochondrial DNA; ROS, reactive oxygen species; SOD, superoxide dismutase.

There are many antioxidants such as superoxide dismutase (SOD) which converts O_2_^−^ to H_2_O_2_; catalase or peroxidase which converts H_2_O_2_ to H_2_O; and glutathione peroxidase, which converts H_2_O_2_, hydroperoxides (R-OOH) and peroxide lipids to H_2_O. Moreover, there are also many non-enzymatic antioxidants, molecules such as vitamins E and C, carotenes, polyphenols, quinones, glutathione and metallic elements, such as selenium, zinc, iron, or copper, among others, which are capable of reducing ROS levels [4]. Under physiological conditions, all antioxidant mechanisms minimize ROS production and therefore act as protective systems against oxidative stress. However, in the presence of mitochondrial dysfunction, ROS generation could increase beyond the detox threshold, compromising the cell viability. In addition, modest endogenous antioxidant defense sensitizes the brain to oxidative stress. That is, comparatively low endogenous antioxidant defense relative to many tissues (e.g., liver) makes the brain susceptible to disrupted redox homeostasis [5]. Beyond the protective endogenous antioxidant enzymatic defense cell mechanisms, the exogenous antioxidants, including those administered through the diet, such as polyphenols, carotenes and vitamins, have been widely described in the literature to play a role in halting free oxygen radicals towards redox balance, also in the context of neurodegeneration [6] (Figure 1).

## 4. Neurodegeneration and Mitochondrial Involvement

Critical roles of the mitochondria in neurons are ATP generation, Ca^2+^ buffering, ROS generation, and antioxidant activity. Neurons are high-demand energy cells closely related to the function, maintenance, and dynamics of mitochondria. In most neurological disorders, mitochondrial activities and dynamics are disrupted, which is associated with high ROS levels, low ATP generation, and apoptosis.

Neurodegenerative diseases, including Alzheimer’s disease, Parkinson’s disease, Huntington’s disease and amyotrophic lateral sclerosis, are a group of heterogeneous disorders, characterized by the progressive loss of specific neuronal populations and impairment of circuits in the central nervous system triggered, at least partly, by mitochondrial impairment [7]. Due to the low potential of neural regeneration, mitochondrial damage results in detrimental effects for neuron survival [8]. Moreover, the accumulation of altered proteins occurring in many neurodegeneration processes may impact on mitochondrial turnover and biogenesis, leading to mitophagy alterations described in neurodegenerative diseases [9]. Such mitochondrial abnormalities lead to increased ROS generation and oxidative stress, ultimately compromising the cell viability. To fight against ROS-mediated oxidative cell damage, a wide range of antioxidant therapeutic approaches mainly targeting mitochondria are available and have been addressed in neurodegeneration processes. This review highlights the antioxidant interventions conducted in neurodegenerative disorders including Alzheimer’s disease, Parkinson’s disease, Huntington’s disease and the less represented amyotrophic lateral sclerosis, while summarizing the latest randomized controlled trials conducted [10]. As we will review, most of these antioxidant interventions conducted on neurodegenerative processes rely on coenzyme Q, melatonin, N-acetylcysteine, resveratrol, polyunsaturated fatty acids such as docosahexaenoic acid (DHA) and eicosapentaenoic acid (EPA), which have the potential to scavenge many ROS (including free radicals, peroxynitrites, hydroxyls, peroxyls, and other nitrous oxides), induce antioxidant enzymes and inhibit pro-oxidant pathways [11].

## 5. Mitochondrial Antioxidant Interventions in Chronic Neurodegeneration

### 5.1. Mitochondrial Antioxidant Molecular Targets in Alzheimer’s Disease

Dysfunction of cell bioenergetics is a common feature of neurodegenerative diseases, such as Alzheimer’s disease. Mitochondrial dysfunction and oxidative stress are prominent functional alterations in Alzheimer’s disease pathophysiology [12]. Disrupted energy utilization implicates mitochondria at its nexus. Classical neuropathological hallmarks of disease (β-amyloid and Tau) and sporadic Alzheimer’s disease risk genes (*APOE*) may trigger mitochondrial disturbance, yet mitochondrial dysfunction may incite pathology. In fact, electron microscopy pictures of Alzheimer’s-disease-affected brains have revealed altered mitochondrial infrastructures [13]. On the other hand, cultured cells and isolated mitochondria maintained in the presence of amyloid beta (Aβ) show reduced electron transport chain enzyme activities [14,15]. Preclinical and clinical efforts have overwhelmingly centered on the amyloid pathway, but clinical trials have yet to reveal clear-cut benefits. Alzheimer’s disease therapies aimed at mitochondrial dysfunction are few and concentrate on reversing oxidative stress and cell death pathways. Novel research efforts aimed at boosting mitochondrial and bioenergetic function offer an alternative treatment strategy. Enhancing cell bioenergetics in preclinical models may yield widespread favorable effects that could benefit persons with Alzheimer’s disease [12]. This is in line with the wide array of compounds have been used to treat Alzheimer’s disease by interfering with mitochondrial metabolism and oxidative stress (Table 1). One example is curcumin, a polyphenol derived from turmeric [16], one of the natural compounds which suppresses tumor necrotic factor (TNF) activity, formation of beta-amyloid plaques and protects brain cells from noxious agents; therefore, this could become a potential effective strategy to treat Alzheimer’s disease [17].

**Table 1:** Antioxidant and mitochondrially targeted neuroprotective drugs in different neuropathological models.

Drug Class (Compound)	Mechanism of Action	Therapeutic Outcomes	References
a-Lipoic acid	Scavenges the toxic by-products of lipid peroxidation	Antioxidant properties in AD	[18,19]
*Bacopa monnieri* extract	Reduces oxidative stress	Ameliorates learning and memory impairments through synaptic protein, neurogranin, pro-and mature BDNF signaling, and HPA axis in PNS in the rat brain	[20]
Carotenoid (Lycopene)	Suppress oxidative damage	Antioxidant, anti-inflammatory, memory enhancing and neuroprotective activities in HD	[18,21]
Cholest-4-en-3-one	Scavenges the toxic by-products of lipid peroxidation	Effective in treating painful diabetic and chemotherapy-induced neuropathies	[18]
Cryptotanshinone (quinoid)	Reduces oxidative stress and inflammation	Anti-apoptotic properties in PD-hiNPCs, significantly reduced cellular apoptosis through mitochondrial restoration (reactive oxygen species and mitochondrial membrane potential). These effects are mediated via the nuclear factor erythroid 2-related factor 2 (NRF2) pathway in PD-hiNPCs.	[22]
Curcumin (volatile oil) (Curcuma longa)	Suppress tumor necrotic factor (TNF) activity, formation of Aβ plaques and protects brain cells from noxious agents	Antioxidant, anti-inflammatory and amyloid disaggregating properties in AD	[17,18,21,23,24]
Cystamine (CYS)	Reduces oxidative stress/apoptosis	Increased BDNF protein levels in mouse frontal cortex, prevention of chronic HAL treatment-induced reduction in BDNF, GSH, and Bcl-xl protein levels, prevention of reduction in neuronal cell viability, BDNF protein levels and apoptosis in ND.	[25,26]
DHA (fatty acid)	Reduces oxidative stress and modulates membrane fluidity	Increased membrane fluidity and non-amyloidogenic processing of APP in AD HEK293 cells, leading to enhanced secretion of sAPPα. This enhanced secretion of sAPPα was associated with substantial protection against apoptosis induced by ER Ca^2+^ store depletion	[27]
Dichloroacetate	Activates the pyruvate dehydrogenase complex and lower cerebral lactate amounts	Neuroprotective activity in HD	[18,28]
Disaccharide (Trehalose)	Inhibits amyloid formation, aggregation of β-amyloid and autophagic activities against aggregation proteins (huntingtin)	Neuroprotective properties in HD	[18,21]
Epigallocatechin-3-gallate (Camellia sinensis)	Stabilize mitochondrial functions like ATP levels	Antioxidant properties in AD	[18,21,23]
Ferulic acid (Smallanthus sonchifolius)	Neuroprotective effect against oxidative stress and cell death induced by Aβ42 oligomers	Antioxidant properties in AD	[18,21,23]
Flavones	Reduces oxidative stress	Neuroprotection was found to be mediated via activation of the anti-apoptotic cell survival proteins of the ERK1/2 and PI3K/Akt pathways in neuroblastoma cell lines.	[29]
Ginsenosides Rg1 and Rg3 (Ginseng)	Suppress Aβ induced neurotoxicity, Aβ associated generation of ROS and cell death	Neuroprotective effect in AD	[18,21,23]
JM-20 (anxiolytic)	Acts through mitochondrial metabolism	Strong antioxidant action and neuroprotective effects against Ca^2+^-induced impairment in rats brain, which are both elicited at the mitochondrial level	[30]
LMWSC (sulfated chitosan is the structural analog of heparin converted to low molecular weight polymer by γ-irradiation)	Lipids	Reduction of the intracellular ROS levels in PD, normalization of antioxidant enzymes, mitigation of rotenone induced mitochondrial dysfunction and apoptosis in neuroblastoma cell lines	[31]
Melatonin	Direct scavenger of many ROS species such as free radicals, peroxylnitrites, hydroxyls, peroxyls, and other nitrous oxides under normal conditions	Antioxidant properties in ND. Protective role against H-89-induced memory impairment in mice brain	[11,32]
Mitoquinone	Produces direct antioxidant action by scavenging peroxyl, peroxynitrite and superoxide ROS	Antioxidant properties in PD	[33]
N-acetylcysteine	Protects against cadmium-induced ROS toxicity marked by reduced mitochondrial membrane potential, high cytoplasmic cytochrome c release, reduced Bcl-2 expression, p53 expression and caspase pathways	Neuroprotective properties	[18,34]
N-acetyl-l-tryptophan (L-NAT)		Neuroprotective in primary motor neurons by inhibition of the secretion of Substance P and IL-1β and mitochondrial dysfunction by inhibiting the release of cytochrome c/Smac/AIF and activation of apoptotic pathways (caspase-1, -9, and -3), as well as proteasomal dysfunction through restoring chymotrypsin-like, trypsin-like, and caspase-like proteasome activity in ALS.	[35]
Naringin, hesperidin and kaempferol (flavonoids)	Exerts protective action against peroxynitrite induced oxidative damage and inhibit nitric oxide synthase (involved in HD)	Anti-inflammatory, antioxidant and neuroprotection in HD	[18]
Nicotinamide	Reduces oxidative stress, acts through mitochondria	Inhibition of ketamine-induced neuro-apoptosis by downregulating Bax, inhibiting cytochrome c release from mitochondria into cytosol, and inhibiting the expression of activated caspase-3	[36]
Olanzapine	An antipsychotic agent with affinity for D1 and D2 dopamine receptors as well as 5-HT2A serotonin receptors	Improved motor symptoms in HD	[18]
Olesoxime	Scavenges the toxic by-products of lipid peroxidation	Antioxidant and neuroprotective activities in NP	[37]
P7C3 (aminopropyl carbazole)	Acts through mitochondria	P7C3 stabilized mitochondrial membrane potential in PD (dopaminergic cell lines), reduced ROS production, and inhibited GSK3β activation, p53 activity, Bax upregulation and cytochrome c release exposed to MPP+, and prevented neuronal loss in the *substantia nigra* (mice brain)	[38], p. 3
Celastrol (Celastrus regelii)	Inhibits nitric oxide synthase (involved in PD and HD)	Anti-inflammatory, antioxidant and neuroprotective activities in PD and HD	[18,21]
Peroxiredoxin	Reduces oxidative stress and apoptosis (via signal-regulating kinase (ASK1)-dependent activation of the c-Jun N-terminal kinase/c-Jun and p38 pro-death pathways)	In vitro and in vivo neuroprotection against 6-OHDA toxicity in DA neurons, and preserved motor functions involving the dopamine system in mouse (PD). PRX2 exhibited antioxidant and anti-apoptotic effects via suppression of apoptosis signal-regulating kinase (ASK1)-dependent activation of the c-Jun N-terminal kinase/c-Jun and p38 pro-death pathways	[39]
Quercetin (flavonoid)	Reduces oxidative stress and inflammatory parameters	Quercetin supplementation decreased the neuronal damage, scavenged the free radicals induced by PCBs and protects PCB-induced apoptosis and oxidative stress in the rat brain.	[40]
Retinoic acid	Acts through the proteasome	A treatment of cultured neuroblastoma cells sets up conditions under which proteasome inhibition, and the resultant accumulation of ubiquitinated proteins, loses its ability to kill the cells (PD)	[41]
Riluzole	Reduces ROS generation via induction of glutathione production	Antioxidant properties in ALS	[42]
Sesamol (Sesamum indicum)	Suppress inducible nitric oxide synthase (iNOS) expression and neuroinflammation in hippocampus neurons	Antioxidant and neuroprotective activities in HD	[18]
Sildenafil (phosphodiesterase type 5 inhibitor)	Acts through cyclic GMP phosphodiesterase	Inhibited nitrosative stress and augmented the levels of LC3, beclin-1, ATG5, p-CREB and BDNF and decreased mTOR levels, as well as augmented p-AMPK in mice spinal cord (MS).	[43]
Steroidal lactones (withaferin A, withanolide A, withanolide D-P) (Withania somnifera)	Improves cognitive functions and restores acetyl cholinesterase enzyme activity	Antioxidant and neuroprotective properties in HD	[18]
Terpene lactones (ginkgolides and bilobalides) and flavonoids (flavonols and flavone glycosides) (Ginkgo biloba)	Stabilize mitochondrial functions like ATP levels and interacts with mitochondrial electron transport chain	Antioxidant and neuroprotective properties in dementia, AD and PD	[18,21,23]
Triterpene saponin (glycyrrhizin) and phenol (isoliquiritigenin) (Glycyrrhiza)	Reduces oxidative stress and damage to brain cells	Antioxidant, anti-inflammatory and neuroprotective properties in dementia, AD and PD	[18,21,23]
Triterpenoid saponins (Bacosides A and B) (Herpestis monniera)	Scavenging of free radicals and improves memory	Antioxidant, anti-stress, antidepressant and useful in HD treatment	[18,21]
Triterpenoid saponins (asiaticoside, asiatic acid and madecassoside) (Centella asiatica)	Reduction in the activity of electron transport chain enzymes and decreased mitochondrial viability	Antioxidant and neuroprotective properties in HD	[18]
VDAC1-derived peptide	Forms the permeability transition pore that further promotes apoptosis (through mitochondria)	Aβ Entry into SH-SY5Y Cells (AD) Is Inhibited by the VDAC1 N-Ter Peptide	[44]
Vitamin C	Maintains the integrity of cellular membranes in mitochondria	Antioxidant and neuroprotective activities in NP	[18]
Vitamin E	Maintains the integrity of cellular membranes in mitochondria	Antioxidant properties in AD	[18,19]
Y27632/NAD+/ZVAD-FMK/resveratrol	Kinase/Caspase	Y27632 and NAD+ exert strong synapto-protective activities whereas zVAD-FMK and resveratrol fail to protect synapses (in primary neuronal cultures from mice brain) (neurodegeneration)	[45]

6-OHDA, 6- hydroxide dopamine; AD, Alzheimer’s disease; ALS, amyotrophic lateral sclerosis; APP, β-amyloid precursor protein; BDNF, brain derived neurotrophic factor; DHA, omega-3 fatty; HAL, haloperidol; HD, Huntington’s disease; hiNPS, human induced neuroprogenitor stem cells; HPA, hypothalamic–pituitary–adrenal; GSK3β, glycogen synthase kinase-3 beta; L-NAT, N-acetyl-l-tryptophan; LMWSC, low molecular weight sulfated chitosan; MPP+, 1-methyl-4-phenylpyridinium; MS, multiple sclerosis; NAD+ nicotinamide adenine dinucleotide; ND, neurodegeneration; PD, Parkinson’s disease; PNS, prenatal stress; RD, retinal degeneration; RNCs, retinal cells; ROS, reactive oxygen species; TH, tyrosine hydroxylase; Y27632, Rho Kinase inhibitor.

Focusing on the latest placebo randomized clinical trials using antioxidant approaches conducted during the past year, resveratrol, carotenoids, omega-3 fatty acids, vitamin E and melatonin supplementations have been used in patients suffering from Alzheimer’s disease (Table 2). Many studies have shown that the widely known non-flavonoid polyphenol resveratrol presents antioxidant, anti-inflammatory, and neuroprotective properties and can decrease the toxicity and aggregation of Aβ peptides in the hippocampus of Alzheimer’s disease patients [46]. In addition, it promotes neurogenesis, prevents blood–brain barrier impairment and prevents hippocampal damage by inhibiting Aβ1–42 from crossing the blood–brain barrier and accumulating in the hippocampus [46,47]. Resveratrol has been recently tested showing significant clinical efficacy in combination with donepezil hydrochloride in Alzheimer’s disease patients by improving their inflammatory parameters, such as TNF-alpha and interleukine-6, as well as cognitive function estimated by mini-mental state examination and Alzheimer’s disease assessment scale cognitive subscale and prognosis (ADAS-Cog) during 2 months of treatment (Table 2) [48]. Beyond polyphenols, carotenoids have been widely described as neuroprotective agents for neurological diseases [49]. Among others, they have been described to play a role in amelioration of clinical symptoms (neurocognitive performance and prognosis) derived from neurodegeneration in Alzheimer’s disease [50]. Interestingly, high plasma α-carotene has been associated with a better global cognition in Alzheimer’s disease patients, including higher semantic memory scores [51]. In addition, carotenoid, together with omega-3 fatty acid and vitamin E supplementation, has been recently described to improve working memory in older adults [52]. Finally, the widely known hormone melatonin and its derived effects have also been recently used in Alzheimer’s disease clinical trials. In the most recent clinical trial, non-rapid eye movement sleep onset related to melatonin administration in Alzheimer’s disease patients has been associated with significant changes in the beta and gamma bands during non-rapid eye movement sleep [53]. However, such findings do not explicitly report any clinical- or cognition-related improvement, probably due to the short-term period of treatment applied (2 nights) (Table 2).

**Table 2:** Randomized placebo-controlled clinical trials in the main neurodegenerative disorders (Alzheimer’s disease and Parkinson’s disease) as well as in amyotrophic lateral sclerosis during the past year.

Molecule	Administration	Sample Size	Time Period	Outcomes	Reference
**Randomized controlled clinical trials in AD during the past year**					
Resveratrol	2 gr daily	*n* = 45 resveratrol *n* = 45 placebo	2 months	Compared with control group, the treated group showed higher MMSE score and lower ADAS-cog score	[48]
				Lower clinical indicators of inflammation (TNF-alpha, IL-6)	
Melatonin	5 mg (two nights)	*n* = 4 melatonin *n* = 4 placebo	2 nights	Significant relative power increase in the theta band and a decrease in relative power and EEG coherences in the beta and gamma bands	[53]
Omega-3 fatty acid, carotenoid and vitamin E	430 mg docosahexaenoic 90 mg eicosapentaenoic acid 10 mg lutein 10 mg meso-zeaxanthin 2 mg zeaxanthin 15 mg vitamin E	*n* = 30 cases *n* = 30 placebo	24 months	Fewer errors in working memory tasks (CANTAB-SWM)	[52]
**Randomized controlled clinical trials in PD during the past year**					
Omega3/6 plus vitamins (A, E, γ-tocopherol)	810 mg eicosapentaenoic acid 4140 mg doxosahexaenoic acid 1800 mg γ-linoleic acid 3150 mg linoleic acid 0.6 mg vitamin A 22 mg vitamin E 460 mg γ-tocopherol	*n* = 20 Neuroaspis group *n* = 20 placebo	30 months	Supplementation delayed disease progression (UPDRS)	[54]
Inosine	1500 mg daily	*n* = 149 inosine group *n* = 149 placebo	24 months	Clinical progression (MDS-UPDRS) and dopamine transporter remained unchanged in the untreated group	[55]
Molecular hydrogen	6.5 (0.1) vol% hydrogen gas in 2 L/min of mixed air or placebo air, twice a day for 1 h (through inhalation)	*n* = 7 hydrogen group *n* = 8 placebo	16 weeks	No significant differences in clinical progression (UPDRS)	[10]
Melatonin	25 mg daily	*n* = 13 melatonin group *n* = 13 placebo	3 months	Significant increase of mitochondrial complex I enzymatic activity and respiratory control ratio	[56]
**Randomized controlled clinical trials in ALS during the past year**					
Edaravone	Intravenous 60 mg/d 10 days in alternating cycle of 10 of 14 days of treatment with 14 days off	*n* = 194 (edaravone plus riluzole) group *n* = 130 riluzole group	14 days	Similar survival probability, similar disease progression, similar time to ventilation	[57]

ADAS, Alzheimer’s Disease Assessment Scale-Cognitive Subscale; AD, Alzheimer’s disease; ALS, amyotrophic lateral sclerosis; CANTAB, Cambridge neuropsychological test automated battery; EEG, electroencephalogram; MMSE, Mini-Mental State Examination; PD, Parkinson’s disease; SWM, spatial working memory task; UPDRS, Unified Parkinson’s Disease Rating Scale.

### 5.2. Mitochondrial Antioxidant Molecular Targets in Parkinson’s Disease

Parkinson’s disease is a neurodegenerative disorder characterized by α-synuclein-positive inclusions (Lewy bodies and Lewy neurites) in neurons and axons of *substantia nigra* and other brain regions, leading to loss of dopaminergic stimulation in the striatum and generating the cardinal symptoms of the disease [58]. 1-Methyl-4-phenyl-1,2,3,6-tetrahydropyridine (MPTP) has been widely used to mimic Parkinson’s disease models through selective degeneration of the *substantia nigra* after systemic administration, with significant impact on the understanding and treatment of Parkinson’s disease [59]. Observations from both experimental models and human Parkinson’s disease provide strong evidence for disruptions in mitochondrial dynamics, bioenergetics defects, complex I inhibition of the electron transport chain, and increased ROS [60,61]. Using this and other models of Parkinson’s disease, a great number of therapeutic molecules targeting mitochondria have been tested and reported in the literature (Table 1). The mitochondrially targeted molecules against oxidative stress may become an effective therapeutic strategy for Parkinson’s disease [33] with the presentation of a wide array of molecules (Table 1). Mitoquinone, a modified ubiquinone conjugated to a triphenylphosphonium, is one of the most studied mitochondrially targeted antioxidants [11] which scavenges ROS, including peroxyl, peroxynitrite and superoxide molecules. Mitoquinone has been shown to present therapeutic effects in Parkinson’s disease models. Coenzyme Q10 has shown beneficial effects in MPTP mice, by reducing damage to the nigrostriatal dopaminergic system [62] through an overall reduction in the progression of disability in the 1200 mg per day group compared to the placebo group, which was 44% at 16 months [62].

Therapeutic effectiveness of mitochondria-targeted antioxidants in MPTP-treated N27 cells decreases toxicity, enhances mitochondrial membrane potential and reduces apoptotic markers [63]. Recent clinical trials in Parkinson’s disease have not led to conclusive results yet [64], although previous studies led to a significant improvement in clinical global impression scale (CGI: 6.1 versus 4.6; *p* = 0.024) [65]. In addition, mitochondrial target tetrapeptides (Sezto-Schiller), which contain an aromatic cationic sequence which results in preferential localization to the inner mitochondrial membrane, have been used to prepare mitochondrially targeted antioxidants [9] and have been associated with neuroprotective properties in an MPTP-treated mouse Parkinson’s disease model by preventing MPP+-induced inhibition of oxygen consumption and ATP production, and mitochondrial swelling [66].

Focusing on the latest placebo randomized clinical trials using antioxidant approaches conducted during the past year, omega-3/6 fatty acids and vitamin supplementations have been used in patients with Parkinson’s disease (Table 2). Nutritional approaches using omega-3 and omega-6 rich formulas with classical antioxidant vitamins including gamma-tocopherol, vitamin A (which is not an antioxidant from a biological point of view, but its pro-vitamin beta carotene is) and vitamin E have been recently used in early Parkinson’s disease [54]. In such randomized, double-blind, placebo-controlled trials, the supplementation as an adjuvant treatment significantly delayed disease progression compared with the placebo group, as estimated by the Unified Parkinson’s Disease Rating Scale (UPDRS) (Table 2) [54]. Beyond the abovementioned classical antioxidants mainly including polyphenols, carotenes, fatty acids and vitamins, the antioxidant therapeutic potential of other different types of molecules such as nucleosides, hydrogen or hormones have been also considered in neurodegenerative disorders and recently assayed in randomized controlled clinical trials, with different outcomes. A randomized, double-blind, placebo and controlled phase 3 clinical trial using the oral nucleoside inosine treatment in early Parkinson’s disease has been conducted [55]. In this study, clinical progression rates were not significantly different between participants randomized to inosine and placebo, as estimated by UPDRS. Secondary outcomes, such as dopamine transporter binding as a biomarker of neuronal integrity, remained unaltered as well in the inosine group (Table 2). In terms of treatment safety, participants randomized to inosine, compared with placebo, experienced fewer serious adverse events (7.4 vs. 13.1 per 100 patient-years) but more kidney stones (7.0 vs. 1.4 stones per 100 patient-years) [55]. On the other hand, and based on previous studies confirming the highly effective antioxidant potential of molecular hydrogen in animal models of Parkinson’s disease [67], hydrogen inhalation has been tested through a randomized double-blind placebo-controlled trial performed in 20 participants fulfilling the Movement Disorder Society clinical diagnostic criteria for Parkinson’s disease, although this study did not reveal any beneficial effects in patients, as estimated by UPDRS [10]. The neuroprotective antioxidant effect of the hormone melatonin has been widely tested in both in vivo and in vitro studies [10,67,68,69]. The most recent double-blind, cross-over, placebo-controlled randomized clinical trial study using melatonin in Parkinson’s disease showed significant decreases of plasmatic levels of lipoperoxides, nitric oxide metabolites, and carbonyl groups in proteins in patients with Parkinson’s disease [56]. In contrast, catalase enzymatic activity significantly increased compared with the placebo group. In addition, mitochondrial complex I enzymatic activity, as well as respiratory control ratio, were increased in the melatonin group, although membrane fluidity remained unaltered between groups. Although the outcomes derived from this study are restricted to a molecular level rather than reporting clinical evidence of neurocognitive improvement, taken together these molecular results suggest the effectiveness of melatonin administration in restoring mitochondrial parameters and reducing oxidative stress markers [56] (Table 2).

### 5.3. Mitochondrial Antioxidant Molecular Targets in Huntington’s Disease

Huntington´s disease is an autosomal dominant hereditary disease characterized by an CAG trinucleotide expansion in the huntingtin gene (chromosome 4) [70] with progressive accumulation of mutant huntingtin, affecting neurons of *striatum*, leading to dysfunction at different cellular levels including mitochondrial dysfunction [71]. Distinct studies investigated the harmful influence of human mutant huntingtin in the apoptotic cascade, specifically by triggering various BH3-only proteins [71]. It has been reported that inhibiting caspase cleavage of huntingtin reduces toxicity and aggregate formation in neuronal and nonneuronal cells [72]. Together, all these proteins are involved in the mitochondrial pathogenesis of Huntington’s disease [73]. Classical neurotoxins malonate and 3-nitropropionic acid have been used to model Huntington’s disease [74]. A wide array of compounds has been used to treat Huntington’s disease by interfering with mitochondria (Table 1). Antioxidants have therapeutic effectiveness in Huntington’s disease [75] (Table 1). Natural compounds extracted from *Withania somnifera*, withaferins and withanolides exerted beneficial effects on cognitive functions and ultimately neuroprotective effects in Huntington’s disease models [18]. Interestingly, withanolide A promoted neuritic regeneration and synaptic reconstruction in other neurodegeneration models [76]. BN-82451, a newer antioxidant, improved motor ability and attenuated neurodegeneration in mouse models of Huntington’s disease [18,77]. Vitamin C and α-lipoic acid also had beneficial effects on motor symptoms and extended survival rates in rodents [77]. Coenzyme Q10, a component of mitochondrial membranes and a free radical scavenger, presents therapeutic effects in Huntington’s disease models [62].

Representative quantitative outcomes using apoptotic inhibitors in neurodegenerative models, including Huntington’s disease, have been summarized (Table 1). Of note, any placebo and controlled randomized clinical trial conducting antioxidant interventions in Huntington’s disease has been reported during the past year.

### 5.4. Mitochondrial Antioxidant Molecular Targets in Amyotrophic Lateral Sclerosis

Amyotrophic lateral sclerosis is a neurodegenerative disorder characterized by progressive muscle weakness derived from upper and lower motor neuron loss. It is thought to be caused by genetic (upwards 20 genes linked), environmental and age-related factors [78]. Mitochondrial dysfunction is one of the earliest pathophysiological events in amyotrophic lateral sclerosis and such mitochondrial alterations occur at multiple levels including mitochondrial respiration and ATP production, calcium handling, dynamics, and apoptotic signaling [42]. Associated mutant proteins of the disease accumulate in mitochondria and cause mitochondrial damage. Marked amyotrophic lateral sclerosis therapeutic strategies have focused on mitochondrial biogenesis, ROS reduction and apoptotic inhibition pathways [18,42]. The classical in vivo genetic model of amyotrophic lateral sclerosis is the transgenic SOD1 mice [79,80]. The administration of N-acetyl-L-tryptophan delayed disease onset, extended survival, and ameliorated deteriorations in motor neuron loss, atrophy and performance in transgenic mice, and suppressed inflammation [81]. The study demonstrated the reduction of cytochrome c/smac/AIF release, increased Bcl-xL levels, and inhibition of caspase-3 activation [81]. Administration of drugs such as coenzyme Q10, dexpramipexole, olesoxime, creatine, all associated with antioxidant properties, as reported [82,83,84,85], showed little success on lifespan whereas creatine shields motor neurons and expands the survival of SOD1 mice by 20% [18,42]. The only drug approved by the Food and Drug Administration for treating amyotrophic lateral sclerosis is riluzole [42], which reduces ROS generation through glutathione production [42], targeting these biological pathways closely related to mitochondrially driven apoptosis (Table 1). Focusing on the latest placebo randomized clinical trials using antioxidant approaches conducted during the past year, edaravone has been administered in patients suffering from amyotrophic lateral sclerosis (Table 2). Edaravone is the first known free radical scavenger, which modulates oxidative damage in various diseases, especially neurodegenerative diseases [86]. In the recent randomized trial using intravenous edaravone therapy for patients with amyotrophic lateral sclerosis, no significant differences were observed in survival probability, time to ventilation and change in disease progression (Table 2) [57,86].

## 6. Mitochondrial Antioxidant Interventions in Acute Neurodegeneration

### Mitochondrial Antioxidant Molecular Targets in Traumatic Brain Injury

In contrast to the chronic neurodegenerative conditions described above, severe traumatic brain injury may result in an acute episode of disordered cerebral blood flow which leads to brain oedema and secondary brain injury mediated through a variety of pathways including neuroinflammatory cascades, glutamate toxicity, mitochondrial dysfunction, energy failure and ROS production. It has also been shown that patients suffering severe traumatic brain injury have a 2–4-times greater chance of developing Alzheimer’s type dementia [87,88].

Traumatic brain injury remains a significant disease burden globally [89,90] and despite more than 50 trials of 31 therapies at a cost of USD 1.1 billion since 1993, there has been a universal failure of therapeutic clinical trials to show benefit in traumatic brain injury [91]. The reason for this is likely to be multifactorial, including significant disease heterogeneity and a failure of current classification systems to capture sufficient detail [92]. Traumatic brain injury research remains a topic of significant research investment and numerous studies have investigated mitochondrial antioxidant molecular targets. A full discussion of these pathways is outside the scope of this review and a comprehensive discussion of animal studies of mitochondrial targets in traumatic brain injury may be found in Hakimina et al. [93].

Despite this large number of preclinical trials, three clinical trials have been investigated clinically. The GSH-amplifying agent and ROS scavenger N-acetylcysteine, which has been shown to have positive effects on neuronal survival, suppressing oxidative stress, reducing apoptosis and exerting an anti-inflammatory effect in traumatic brain injury [93] was investigated in a randomized double blind placebo controlled trial in serviceman suffering mild traumatic brain injury following blast injuries in a combat zone, concluding that N-acetylcysteine improved both neuropsychological and medical symptoms following blast injury [94]. Administration of the flavonoid Enzogenol (a herbal supplement) to patients suffering mild traumatic brain injury demonstrated an improvement in cognitive symptoms compared to a placebo in a pilot randomized clinical trial [95]. The antioxidant vitamins C (ascorbic acid) and E (α-tocopherol) were investigated in a randomized double-blind placebo-controlled trial in patients suffering severe traumatic brain injury. The vitamin C group demonstrated a statistically significant (*p* = 0.01) reduction in radiological perilesional oedema, and the vitamin E group demonstrated a statistically significant (*p* = 0.04) reduction mortality and improved Glasgow Outcome Scale outcomes at discharge [96]. Two of these trials represent dietary supplements (Enzogenol and vitamin C and E) and may not be strictly considered as therapeutic drug trials. The trial of N-acetylcysteine is clearly a drug trial but was limited by its application to military servicemen suffering mild symptoms caused by blast injuries. However, they demonstrate the potential for mitochondrial antioxidant therapies in traumatic brain injury.

## 7. Conclusions

Herein we reviewed the most relevant antioxidant interventions in neurodegeneration contexts (Alzheimer’s disease, Parkinson’s disease, Huntington’s disease, and amyotrophic lateral sclerosis) and also traumatic brain injury, while highlighting the latest randomized controlled clinical trials in the past year [10].

It is of note that the current antioxidant approaches through randomized clinical trials in neurodegeneration have been mainly tested as adjunctive treatments included within therapeutic schedules rather than as exclusive interventions [48,97].

Most of the antioxidant agents recently tested in the randomized controlled clinical trials herein discussed belong to polyphenols, carotenes, fatty acids and vitamins family, through exogenous adjunctive interventions. However, the antioxidant and neuroprotective power is not only restricted to these groups, since other antioxidant molecules such as nucleosides, hydrogen gas or hormones have also been tested in clinical trials against neurodegenerative disorders, and mainly in Parkinson’s disease during the last year [55], [10]. The hormone melatonin is one of the most recurrent antioxidant molecules that has been tested in a huge number of diseases, including neurodegenerative disorders. From the most recent randomized clinical trials conducted in the past year and using antioxidants in neurodegenerative diseases found in this review, two of them used melatonin in different neurodegenerative contexts, including Alzheimer’s disease and Parkinson’s disease. Both of them reported interesting significant changes among groups [53,56], such as a significant decrease of lipoperoxides, nitric oxide metabolites, and carbonyl groups in proteins in plasma samples from patients with Parkinson’s disease receiving melatonin compared with the placebo group. Conversely, catalase activity was increased significantly in comparison with the placebo group. Compared with the placebo group, the melatonin group also showed significant increases of mitochondrial complex 1 activity and respiratory control ratio.

There is a huge limitation and set-back in the discovery of an effective drug candidate due to the failed clinical trials. Globally, not all the recently reported randomized clinical trials showed significant changes derived from antioxidant administration. On the contrary, some of them revealed no beneficial effects in the patients [10], or no clinical effects associated with an improvement of the disease [53]. Lack of evidence of improvement related to antioxidant interventions can also occur in widely used therapies. Such is the case of resveratrol, which failed to confer neuroprotection in primary neuronal cultures from mice brains [45]. Thus, not all the findings linked to antioxidant targets report evidence of an amelioration. Nevertheless, those reporting significant changes derived from exogenous antioxidant intake showed promising findings including changes in cognitive function and clinical prognosis [48]. Of note, some of them reported not only clinical amelioration but also improvement of molecular biomarkers, mainly mitochondrial-related activity and oxidative stress parameters [56].

Environmental factors such as exercise and diet have been widely described to play a relevant role in reducing oxidative stress levels through a redox equilibrium. In line with this, Mediterranean diet neuroprotective effects have also been recently depicted through a single center, randomized controlled trial in Parkinson’s disease patients [98]. Interestingly, the Mediterranean diet significantly increased serum total antioxidant capacity and reduced disease severity in Parkinson’s disease patients [98]. In addition, circulating α-carotene levels were associated with higher global cognition scores in a population at risk for cognitive decline in the Mediterranean-DASH Intervention for Neurodegenerative Delay trial [51].

As described, antioxidant therapies have been shown to play a role in neuroprotection [99]. Strengthening this hypothesis but for the other way around, neuroprotective established therapies have been shown to present antioxidant properties. Such is the case of nonergoline dopamine agonists pramipexole and ropinirole in the treatment of Parkinson’s disease [97].

## Data Availability

Not applicable.
